# Patient Satisfaction and Symptoms Improvement in Women Using a Vginal Pessary for The Treatment of Pelvic Organ Prolapse

**DOI:** 10.25122/jml-2019-0042

**Published:** 2019

**Authors:** Nahid Radnia, Maryam Hajhashemi, Tahereh Eftekhar, Maryam Deldar, Taraneh Mohajeri, Samira Sohbati, Zinat Ghanbari

**Affiliations:** 1. Department of Obstetrics & Gynecology, Faculty of Medicine, Hamadan University of Medical Sciences, Hamadan, Iran; 2. Department of Obstetrics & Gynecology, Faculty of Medicine, Isfahan University of Medical Sciences, Isfahan, Iran; 3. Department of Obstetrics & Gynecology, Vali-e-Asr Hospital, Tehran University of Medical Sciences, Tehran, Iran; 4. Department of Obstetrics & Gynecology, Mashhad Islamic Azad University of Medical Sciences, Mashhad, Iran; 5. Department of Obstetrics & Gynecology, Kerman University of Medical Sciences, Kerman, Iran

**Keywords:** Pessaries, Pelvic Floor, Pelvic Organ Prolapse

## Abstract

Pelvic organ prolapse is a common complaint among older women. Vaginal pessary insertion is an appropriate treatment as a non-surgical method with few complications. This paper is a prospective observational study of 68 patients with pelvic organ prolapse that was carried out at the Imam Khomeini Hospital’s Pelvic Floor clinic. The degree of pelvic organ prolapse was graded according to the Pelvic Organ Prolapse Quantification (POP-Q) System. For all patients, the Pelvic Floor Distress Inventory-20 (PFDI-20) questionnaire was completed before vaginal pessary insertion, and after approximately 6 months of treatment. After 6-8 months, we found out that vaginal discharge was significantly increased and the feeling of fullness in the vagina was significantly decreased. However, sexual dissatisfaction, the feeling of incomplete evacuation, fecal and urinary incontinence, frequent urination, and pain or discomfort in the genital region were not significantly different after using a pessary. Approximately half a year later, 96.7% of the women with a successful pessary fitting trial were satisfied and reported a significant improvement in symptoms. Further studies with larger sample size, a different type of pessary, and a longer follow-up duration are recommended to evaluate all the symptoms associated with pelvic organ prolapse and its treatment.

## Introduction

Pelvic organ prolapse (POP) is the herniation of the pelvic organs to or beyond the vaginal walls. Most women with POP experience symptoms that impact their quality of life such as sexual function, daily activities and exercise [1].

The pelvic structures that may be involved in pelvic organ prolapse include the uterus (uterine prolapse) or vaginal apex (apical vaginal prolapse), anterior vaginal wall (cystocele), or posterior vaginal wall (rectocele).

Anatomic support of the pelvic organs in women is provided by an interaction between the muscles of the pelvic floor, like the levator ani muscle complex, and connective tissue attachments within the bony pelvis. All levels of vaginal support are connected through a continuous endopelvic fascia support network; these structures stabilize the pelvic organs in the correct position [2, 3].

Pelvic organ prolapse may be symptomatic or asymptomatic. Proven risk factors for POP include parity, advanced age, chronic constipation and obesity [4, 5]. Treatment methods for pelvic organ prolapse have not been completely studied. There are two basic patterns for treating POP, including conservative treatment and surgery. Surgical repair is one approach to POP treatment. However, some women may prefer to avoid surgical treatment or may not be candidates for surgery [21]. Vaginal pessaries are intravaginal support devices that may be controlled by the patient. These devices reduce prolapse or incontinence [7] and are an alternative non-invasive treatment option with rapid symptom relief without surgical complications for women with these conditions. Pessary use is the most cost-effective treatment alternative for treating POP [22]. Some evidence suggests that women with POP who use vaginal pessary have a lower stage of prolapse on clinical examinations after pessary insertion [8]. Patient’s acceptance of pessaries varies from 42 to 100 percent [9–12] and is related to appropriate counseling and encouragement from the physician [13]. In this study, we evaluated the satisfaction and symptom improvement of patients using a vaginal pessary in order to treat POP.

## Materials and Methods

Women with symptomatic pelvic organs prolapse referred to the Pelvic Floor clinic at Imam Khomeini Hospital, and they were advised to use a vaginal pessary, the physician describing the advantages and disadvantages to the patients.

The 68 patients with pelvic organ prolapse, who met the inclusion criteria (pelvic organ prolapses with POP-Q stage ≥ 2 in one or more components, patients who preferred conservative therapy to surgery and patients who had no active pelvic infection) and agreed to try a vaginal pessary were included in the study. After the patients underwent pessary insertion, long-term follow-up was carried out. Also, vaginal estrogen was prescribed for all patients included in this study.

At the first visit, demographic data were collected, and all patients were examined. The degree of pelvic organ prolapse was graded according to the POP–Q test. For all patients, the Pelvic Floor Distress Inventory-20 (PFDI-20) valid questionnaire was completed during the first visit and follow-up.

Approximately 6 months (from 6 to 8 months) after pessary insertion, the patients came back to the clinic, and the POP–Q test was performed. The Patient Global Impression of Improvement (PGI-I) valid questionnaire was used to assess patient satisfaction after pessary insertion.

PGI-I is a single question and global index transition scale that is asking the patient to rate their urinary tract condition and the response of a condition to a therapy (transition scale). It is simple, direct, yet easy to use and intuitively understandable to clinicians. Additionally, PGI-I has excellent construct validity [14].

There are different types of vaginal pessaries that we used for different patients as needed (ring with support, Gelhorn and cube pessary). In all cases, we used a type of pessary that was appropriate to the patient’s needs and associated conditions.

During follow-up examination, pessary placement was first checked after 1 or 2 weeks. If the patient was comfortable and satisfied with the pessary, a next follow-up visit was scheduled at 6 months.

All patients completed the PGI-I questionnaire again during the follow-up visit in the Pelvic Floor clinic before the examination and removal of the pessary. The average 6-month responses were compared with the baseline responses.

T-test was used to compare the means in normal data while the Mann-Whitney U test was used to analyze abnormal data. Statistical analysis was carried out using SPSS 20. P values of <0.05 were considered statistically significant

## Results

Of the 68 patients with symptomatic pelvic organ prolapse (stage ≥2 POP-Q) with a mean age of 68.9 years (ranging from 34 to 89 years) who met the inclusion criteria, 60 patients (88%) successfully retained the pessary approximately 6 months after insertion. Two patients could not retain the pessary and six patients left the study and underwent surgery.

In our study, six (9%) patients had a history of prolapse surgery, six (9%) patients had a history of hysterectomy, 29 (42.6%) patients were sexually active, 8 (12.1%) patients tended to undergo surgery at the first visit and 66 (97.1%) patients were postmenopausal Table 1.

**Table 1:** Patient Characteristics (number= 68)

Age (y): mean(SD)	68.98(10.154)
**Menopause, n (%)**	97.1%
**Previous hysterectomy (percent)**	10.3%
**Previous prolapse surgery (percent)**	8.8%
**Sexually active**	42.6%
**Tendency to surgery before pessary**	12.1%

At the first follow-up visit, 30% of patients reported significant vaginal discharge and cervical erosion after pessary insertion (P<0.001), which improved relatively at the 8-month follow up.

At the first visit, the PDFI-20 questionnaire was completed by all patients. 34 patients complained of pelvic organ prolapse and 10 patients complained of colorectal and anal symptoms. 17 patients had a feeling of fullness in the vagina, 25 patients experienced frequent urination, and 18 patients experienced urgency, some patients had urine leakage(as wet urgency) and some patients didn’t have leakage until urination occure (dry urgency).

In order to determine POP-Q status before pessary insertion, three measurements were taken: the vaginal apex and the anterior and posterior vaginal walls. In all patients, the results corresponded to stage 3 or higher.

**Figure 1:**
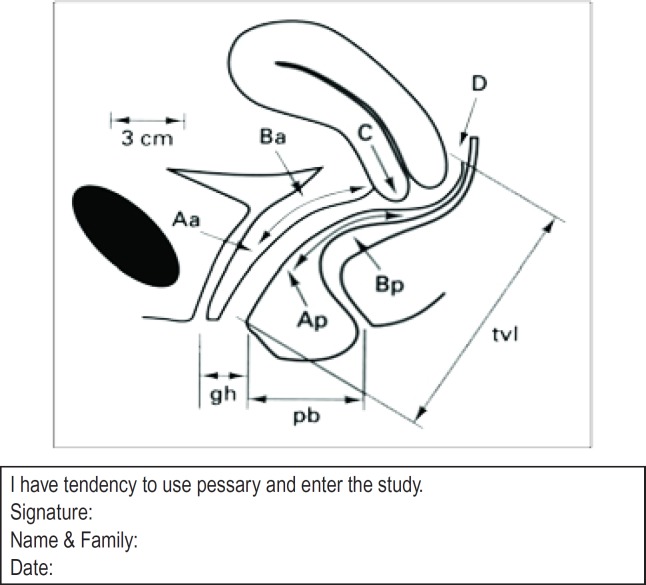
POP-Q points

Follow-up was carried out little after pessary insertion and approximately 6 months after the intervention and the feeling of fullness or pressure in the vagina, as well as associated symptoms, were improved significantly (P-value < 0.001), in all points except pb (perineal body) and tvl (total vaginal length) points (Figure 1).

After approximately 6 months, the vaginal discharge was significantly increased, while the feeling of fullness in the vagina was significantly decreased (P-value < 0.001).

The results of our study showed that sexual dissatisfaction in patients and their partners, the feeling of incomplete evacuation, fecal and urinary incontinence, frequent urination, and pain or discomfort in the genital region were not significantly different after using a pessary Table 2.

**Table 2:** Baseline symptoms and change of symptoms after insertion of vaginal pessaries at approximately 6 months.

Symptom	Before pessary		Change of symptoms	
		Better	Worse or New created	No change
**Feeling of fullness in the vagina**	94.9%	97.6%	0%	2.4%
**Vaginal soreness and discharge**	8.6%	0%	50%	50%
**Urge bowel incontinence**	12.7%	10%	5%	85%
**Incomplete bowel evacuation**	19%	13.6%	4.5%	77.3%
**Stress urinary incontinence**	34.4%	50%	3.1%	46.9%
**Urge urinary incontinence**	53.3%	36.1%	8.3%	55.6%
**Incomplete bladder emptying**	35.6%	42.4%	15.2%	42.4%
**Pain**	3.4%	0%	28%	72%
**Lack of sexual satisfaction**	12.5%	27.3%	9.1%	63.6%

After approximately 6 months, 96.7% of the women with a successful pessary fitting trial were satisfied and had significance symptoms improvement Tables 3, 4.

**Table 3:** Satisfaction after pessary use

Satisfaction		Frequency	Percent
**Valid**	Very satisfied	50	82.0
	Satisfied	5	8.2
	No change	5	8.2
	Dissatisfied	1	1.6
**Total**		61	100.0

**Table 4:** Symptom improvement after pessary insertion at approximately 6 months

Symptom improvement		Frequency	Percent
**Valid**	So much better	50	82.0
	Much better	3	4.9
	Better	2	3.3
	No change	5	8.2
	So much worse	1	1.6
	Total	57	93.4
**Total**		61	100.0

## Discussion

Pelvic organ prolapse is a common complaint in postmenopausal and older women in the entire world [15].

In particular situations, such as the presence of severe medical comorbidities that make the patient a poor surgical candidate, the preference for nonsurgical treatment or the need to delay surgery for several weeks or months, patients suffering from recurrent pelvic organ prolapse or stress urinary incontinence are recommended to use a vaginal pessary.

The use of a pessary, although having many benefits, also has a few disadvantages that limit its use in patients with local infections. Also, sexually active women who are unable to remove and reinsert the pessary are not appropriate candidates for pessary insertion the most important advantages and disadvantages of pessary mentioned in Table 5.

**Table 5:** POP-Q test before and after pessary insertion

		Mean	N	Std. Deviation	P-value
Pair 1	Aa_0	1.800	60	2.0630	**.000**
	Aa_1	–1.225	60	1.4451	
Pair 2	Ba_0	3.617	60	2.8394	**.000**
	Ba_1	–1.050	60	1.5642	
Pair 3	C_0	3.900	60	3.5052	**.000**
	C_1	-3.592	60	2.8351	
Pair 4	GH_0	5.983	59	5.0026	**.011**
	GH_1	4.186	59	1.4707	
Pair 5	Pb_0	3.458	59	.9882	**.093**
	Pb_1	3.653	59	1.1790	
Pair 6	TVL_0	8.780	59	1.6302	**.477**
	TVL_1	8.932	59	1.5769	
Pair 7	Ap_0	–.133	60	2.1110	**.001**
	Ap_1	–1.283	60	1.4391	
Pair 8	Bp_0	.642	60	2.8509	**.000**
	Bp_1	–1.075	60	1.5940	
Pair 9	D_0	.690	51	6.9379	**.000**
	D_1	–3.912	51	2.6262	

There are several studies proving that vaginal pessary improves some of the symptoms of pelvic organ prolapse [16].

In a prospective study that Clemons et al. carried out in 2004,17.100 women with symptomatic pelvic organ prolapse were fitted with a pessary, and 73 women had a successful 2-week pessary fitting trial. After approximately 6 months, 92% of women with a successful pessary fitting trial were satisfied. Dissatisfaction in 8% of patients was because of occult stress incontinence. Nearly all symptoms resolved; 50% of the urinary symptoms improved, stress incontinence improved in 45% of patients and was a common side effect in other patients.

However, in Clemons’ study, unlike our study, a valid questionnaire was not used to assess other symptoms like bowel and bladder symptoms. After evaluating the questionnaire, we found out that vaginal discharge and the feeling of fullness in the vagina were significantly decreased at approximately 6 months after the intervention, but other symptoms like urinary incontinence did not change significantly. It is essential to have a proper questionnaire to evaluate the symptoms before and after pessary insertion.

In another prospective observational study conducted in 2016, 18 women with pelvic organ prolapse presenting for a vaginal pessary fitting were asked to complete the ICIQ-VS questionnaire (International Consultation on Incontinence Modular Questionnaire-Vaginal Symptoms), and their satisfaction was evaluated using the visual analogue scale (VAS) prior to pessary fitting, and after 3 and 6 months of treatment. Forty women entered this study, and all vaginal symptoms and the quality of life scores significantly improved after 3 and 6 months of treatment. The use of a vaginal pessary for up to 6 months improved the vaginal symptoms, quality of life and satisfaction in women with pelvic organ prolapse.

**Satisfaction form of Patients with pelvic organ Prolapse**

**Advantages:**

– Reversible

– Having no serious complication

– At any time can be removed in case of dissatisfaction

– Whenever a patient wants, can remove it and get surgery

– Alternative procedure instead of surgery and have no surgical problems

**Disadvantages:**

– The need for follow up in the specified intervals for the patient’s and frequent examination

– Urinary incontinence following pessary insertion

Although some patients had some complications like the erosion of the vaginal wall or vaginal discharge, these complications were improved by adequate lubrication and vaginal estrogen treatment.

One strength of this study was that patients were treated with pessaries over 6 months, and followed-up for 3 and 6 months after pessary insertion.

However, in this study, a validated Thai version of the ICIQ-VS and a simple VAS tool were used to interpret patient’s satisfaction, while we used the Patient Global Impression of Improvement (PGI-I) questionnaire to assess satisfaction regarding pessary insertion. Also, the stage of pelvic organ prolapse was assessed by a single examiner that performed the POP-Q exams.

One of the weaknesses of this study, unlike our study, was its small sample size.

Similar studies have shown that most women with pelvic organ prolapse tend to use a pessary as a non-invasive treatment [19,20].

Pelvic organs prolapse is one of the most common complaints in older women [21].

Due to the risk of surgery in patients, especially in the elderly, our first recommendation is pessary treatment. Our study showed that pessaries improve POP in the majority of patients and can replace surgery in some patients, especially elderly patients.

The pessary should be noted in patients with many contraindications and high risk of surgery. Also, the pessary is an appropriate treatment option in patients with a history of medical problems such as heart disease, high blood pressure, cerebrovascular disease, and history of myocardial infarction.

Also, further studies are recommended to focus on other prolapse symptoms with different types of pessaries in order to assess the response to treatment.

## Acknowledgment

We wish to thank Miss Mahnaz Alizadeh and colleagues at the Clinical Research and Development Unit | of Fatemieh Hospital of Hamadan who provided insight and expertise that greatly assisted the research. We also gratefully acknowledge Mrs. Lotfi and the professors of the Pelvic Floor clinic of Imam Khomeini Hospital in Tehran that helped with the manuscript.

## Conflict of interest

The authors declare there is no conflict of interest.
